# Adipose stromal cells bioproducts as cell-free therapies: manufacturing and therapeutic dose determine in vitro functionality

**DOI:** 10.1186/s12967-023-04602-9

**Published:** 2023-10-16

**Authors:** Renata Skovronova, Eleonora Scaccia, Sandra Calcat-i-Cervera, Benedetta Bussolati, Timothy O’Brien, Karen Bieback

**Affiliations:** 1 Department of Molecular Biotechnology and Health Sciences, University of Turin, Turin, Italy; 2 Institute of Transfusion Medicine and Immunology, Medical Faculty Mannheim, Heidelberg University, German Red Cross Blood Service, Baden-Württemberg—Hessen, Friedrich-Ebert-Str.107, 68167 Mannheim, Germany; 3 College of Medicine, Nursing and Health Science, School of Medicine, Regenerative Medicine Institute (REMEDI), University of Galway, Galway, Ireland; 4 CÚRAM, SFI Research Centre for Medical Devices, University of Galway, Galway, Ireland; 5 Mannheim Institute of Innate Immunoscience, Medical Faculty of Mannheim, Heidelberg University, Mannheim, Germany

**Keywords:** Mesenchymal stromal cells, Secretome, Immune modulation, Angiogenesis

## Abstract

**Background:**

Extracellular vesicles (EV) are considered a cell-free alternative to mesenchymal stromal cell (MSC) therapy. Numerous reports describe the efficacy of EV in conferring immunomodulation and promoting angiogenesis, yet others report these activities to be conveyed in EV-free bioproducts. We hypothesized that this discrepancy may depend either on the method of isolation or rather the relative impact of the individual bioactive components within the MSC secretome.

**Methods:**

To answer this question, we performed an inter-laboratory study evaluating EV generated from adipose stromal cells (ASC) by either sequential ultracentrifugation (UC) or size-exclusion chromatography (SEC). The effect of both EV preparations on immunomodulation and angiogenesis in vitro was compared to that of the whole secretome and of the EV-free protein fraction after SEC isolation.

**Results:**

In the current study, neither the EV preparations, the secretome or the protein fraction were efficacious in inhibiting mitogen-driven T cell proliferation. However, EV generated by SEC stimulated macrophage phagocytic activity to a similar extent as the secretome. In turn, tube formation and wound healing were strongly promoted by the ASC secretome and protein fraction, but not by EV. Within the secretome/protein fraction, VEGF was identified as a potential driver of angiogenesis, and was absent in both EV preparations.

**Conclusions:**

Our data indicate that the effects of ASC on immunomodulation and angiogenesis are EV-independent. Specific ASC-EV effects need to be dissected for their use as cell-free therapeutics.

**Supplementary Information:**

The online version contains supplementary material available at 10.1186/s12967-023-04602-9.

## Background

Mesenchymal stromal cells (MSC) are considered one of the most promising adult stromal/stem cell types in regenerative cell-based therapies. While MSC were first isolated from bone marrow aspirates, other sources such as the adipose tissue have become excellent alternatives due to accessibility and ease of sourcing [1]. These properties intensified the number of preclinical and clinical studies showing the role of adipose stromal/stem cells (ASC) in tissue regeneration [2]. These pro-regenerative effects are not restricted to cell-to-cell effects but can also be reproduced by the secretome of MSC, a wide compendium of bioactive molecules secreted into the extracellular space [1]. The secretome can be broadly divided into two fractions: extracellular vesicles (EV) and a wide variety of soluble proteins, lipids, and free nucleic acids [3].

EV are a heterogeneous population of lipid bilayer-delimited particles classified according to their size (< 200 nm or > 200 nm) [4] and biogenesis [5]. EV are an integral component of the cell-to-cell communication network, containing a wide range of proteins, lipids, and several coding and noncoding nucleic acids [6, 7]. Upon transfer to the receiving cells, they can modulate downstream signaling pathways through the direct stimulation of cell surface receptors or by the direct transfer of bioactive molecules [4, 8]. On the other hand, the protein-rich fraction of the secretome includes different growth factors and cytokines with key roles in the regenerative activity such as vascular endothelial growth factor (VEGF), transforming growth factor β (TGF-β), or hepatocyte growth factor (HGF), among others [9, 10].

The use of the MSC-derived secretome is considered a promising approach in the cell therapy field: Besides the ease in storage and transportation as cell-free product, the administration of bioactive factors as a therapeutic product represents a safer alternative, without the risks of tumor development or emboli formation, entrapment in lung microvasculature, and less immunogenicity [11]. Additionally, dissecting the mechanisms of action of specific factors within the secretome may allow to bioengineer and upscale production of these paracrine factors [12, 13].

Since the first studies indicating that the secretome could replicate the cardioprotective effects of MSC transplantation [14], a plethora of preclinical evidence has supported the beneficial role of MSC bioproducts in tissue regeneration [15–17]. More recently, MSC derived EVs have been successfully used in clinical studies as a novel treatment for e.g. graft-versus host disease, diabetic nephropathy and severe SARS-CoV-2 infection [18–21].

While the MSC-secretome may be a substitute for cell therapy, several challenges need to be addressed to standardize it as a product. In fact, it presents numerous difficulties, as cellular secretions are generated by a dynamic and complex process that varies according to the tissue from which the cells are isolated, the donors and in response to different culture conditions [12, 22, 23].

Lack of consensus within the field also exists in respect of EV isolation strategies: precipitating agents, ultracentrifugation, tangential flow filtration, and size exclusion chromatography may result in differences in the obtained product [24, 25]. Recent studies have indicated that the method of EV isolation can affect the efficiency and generate potential confounding factors leading to the misattribution of beneficial properties [26].

**Fig. 1:**
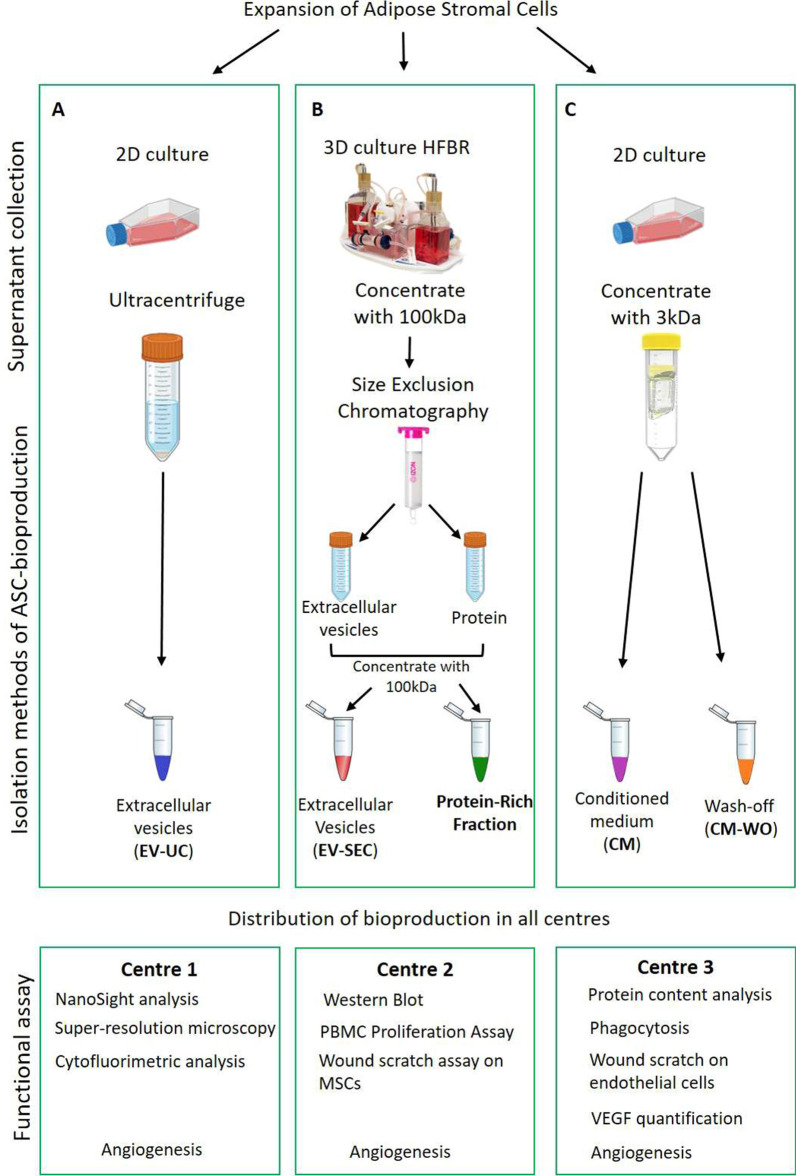
Scheme of isolation of ASC bioproducts. The ASC were cultured in traditional 2D culture flasks in serum-free medium for the production of EV-UC (**A**). The supernatant was collected and then ultracentrifuged for 2 h at 100,000 × *g*, the EV-UC were collected, cryopreserved and shared between the centers. For the production of EV-SEC (**B**), the ASC were cultured in a 3D hollow fiber bioreactor (HFBR), from which the supernatant was collected and concentrated with 100 kDa filter. The samples were then processed with size exclusion chromatography and the EVs and protein fraction were concentrated with a 100 kDa filter and collected. The EV-SEC and the protein-rich fraction were shared between centers. Finally, the ASC were cultured in traditional 2D culture flasks, the supernatant was collected and concentrated with 3 kDa and the conditioned medium and wash-off were collected during the process (**C**), cryopreserved and shared between centers

Despite the numerous studies indicating the beneficial effects of EVs, we previously failed to reproduce such potential [27, 28]. In the present study, we sought to deep dive the differential effects of adipose stromal cell (ASC) bioproducts, analyzing different EV production and isolation methods as well as the complete secretome or the protein-rich fraction in a multicenter comparative study (Fig. 1).

## Material and methods

### Cell culture

ASC from lipoaspirates from three donors were processed by the group of Prof. Karen Bieback (University of Heidelberg, Heidelberg, Germany) after informed consent. The Mannheim Ethics Commission II approved the study (vote 2011-215 N-MA). The ASC were cultured using MEM-⍺ media, Gibco, ThermoFisher Scientific, 2,561,029) and 10% Fetal Bovine Serum (FBS, 10270-106, Gibco, MA, USA) at 37 ℃ with 5% CO_2_ and controlled humidity. These three ASC batches (referred to a N = 3 biological replicates in the figure legends) were shipped as cryo-aliquots to the other two centers to be cultured under identical harmonized culture conditions from passage 4–6 as detailed previously [29]. Bioproducts were derived from the conditioned medium of either 3D cultured ASC, processed by size exclusion chromatography to yield (1) EV-SEC or the (2) protein-rich fraction, or 2D-cultured cells, processed by ultracentrifugation to yield (3) EV-UC or after concentration to yield (4) the conditioned medium (CM) or (5) the respective wash-off (CM-WO) (Fig. 1).

### 3D culture of ASC

At 80% confluence, ASC were passaged and seeded in a hollow-fibber bioreactor at a concentration of 14 × 10^6^ cells/cartridge (20 kDa MWCO, 450 cm^2^, C2025D, FiberCell System-KD Bio, France). Prior to injecting the cells, a ‘pre-culture step’ was carried out to initiate and activate the bioreactor, first Dulbecco’s phosphate-buffered saline (PBS) for 24 h, followed by fibronectin coating over-night. After the pre-culture process, ASC were seeded in serum-free MEM-⍺ in the extra-capillary space, at 37 ℃ with 5% CO_2_ and controlled humidity for 7 days without harvesting the supernatant, with continuous monitoring of glucose levels. Serum-containing medium was used as circulating medium, given that EVs and high molecular weight proteins cannot cross the 20 kD MWCO filter fiber and thus do not contaminate the cell-derived EVs harvested from the extra-capillary-space (according to the “Hollow Fiber Bioreactor Protocol for Mesenchymal Stem Cells” by fibercellsystems.com) ASC were cultured for 4 weeks in the bioreactor and during this period, the supernatant was collected daily. Following centrifugation to remove cell debris (5 min at 420 × g), the supernatant was stored at – 80 ℃ until EV isolation by size exclusion chromatography (see below) was performed. Cells were harvested and counted to calculate the bioproduct per producer cell concentration.

### Conditioned media collection

The secretome obtained *in* vitro, also named conditioned media (CM), was generated from ASC at passage 4 to 6. Upon reaching 80% confluence, cells were washed with PBS and incubated for 24 h in serum-free MEM-⍺ medium. The supernatant was collected and centrifuged for 5 min at 400 × *g* to remove cell debris before being placed in centrifugal concentrator units of 3 KDa molecular weight cut-off (UFC9003, Merck Millipore, USA). The CM was centrifuged for 90 min at 3,000 × *g*, 4 ℃ using an Eppendorf^™^ 5810 R Centrifuge to achieve tenfold concentration. The flow-through resulted from the concentration step (thereafter named wash-off, CM-WO) was kept and used as a control. Concentrated conditioned media samples were stored at – 80 ℃ until further use. Cells were harvested and counted to calculate the bioproduct per producer cell concentration.

### EV Isolation by ultracentrifugation

When the cells reached 80% confluence, they were starved for 16–24 h in serum-free medium. The supernatant was collected and centrifuged for 20 min at 3000 × *g* to remove cell debris and apoptotic cells. The supernatant was then ultracentrifuged for 2 h at 100,000 × *g*, 4 ℃ using Beckman Coulter Optima L-100 K Ultracentrifuge (Beckman Coulter, CA, USA) with the rotor type 70Ti. The EV pellet was resuspended in PBS supplemented with 1% DMSO. The suspension of EVs (EV-UC) was then stored at − 80 ℃ until further use. EVs were collected from ASC at 4-6th passage. Cells were harvested and counted to calculate the cell equivalents used for cell treatments.

### EV Isolation by size-exclusion chromatography

After thawing at 4 ℃, samples were centrifuged for 10 min at 300 × *g* and 20 min at 4000 × *g*. After, the supernatant was filtered through a 0.2 µm syringe filter and concentrated with a 100 kD MWCO concentration filter to a final volume of 10 mL. The qEV10-IZON column 35 mm was initially washed with sterile PBS, and then 10 mL of the sample was added to concentrate it to the final volume of 1.5 mL (Vivaspin 20, 100,000 MWCO PE, Sartorius). Each EV sample (EV-SEC) and the resultant supernatant containing the protein fraction (Protein-Rich Fraction) were collected, concentrated (Vivaspin 20, 100,000 MWCO PE, Sartorius) and stored at – 80 ℃ until further use.

### EV/CM treatment

ASC derived bioproducts were used at a ratio of 2:1 and 20:1 relative to recipient cells. To do so, we counted the number of ASC after harvesting and used it to relate the number of particles/volumes generated of EVs and CM respectively for each bioproduct.

### Nanotracking analyses

After the isolation, the concentration of all the samples was measured (a) by Nanosight NS300 or (b) ZetaView.

### Nanosight analysis

After the isolation, the concentration of all the samples was measured (a) by Nanosight NS300 (Malvern Instruments Ltd., Malvern, UK) equipped with a 488 nm laser module that utilizes Brownian motion and refraction index. The particle size scatters 10 nm to 1000 nm, although the optimized size range is 70–300 nm. It uses the scattered light to detect a particle and tracks its motion as a function of time. The particles’ scattered light was recorded with a light-sensitive camera under a 90^°^ angle to the irradiation plane. This angle allows the Brownian motion of the EVs. Samples were diluted 1:100 in physiologic solution. For each sample, 3 videos of 60 s at camera level 15 and threshold 5 were captured using a syringe pump 30. All the samples were characterized with NTA 3.2.16 Analytical software. The NTA settings were kept constant between samples.

### ZetaView analysis

After the isolation, the concentration of all the samples was measured b) by ZetaView (Particle Metrix GmbH, Germany). 1 μL of concentrated EVs was diluted in sterile-filtered PBS in a dilution 1:1,000 and visualized using the ZetaView (sensitivity 80%, shutter 100, 11 positions, 2 cycles; Particle Metrix, Germany).

### Super-resolution microscopy

Super-resolution microscopy pictures of EVs were obtained using a temperature-controlled Nanoimager S Mark II microscope from ONI (Oxford Nanoimaging, Oxford, UK) equipped with a 100 ×, 1.4NA oil immersion objective, an XYZ closed-loop piezo 736 stage, and 405 nm/150 mW, 473 nm/1 W, 560 nm/1 W, 640 nm/1 W lasers and triple emission channels split at 640/and 555 nm. For sample preparation, we followed the manufacturer’s protocol using EV profiler Kit ONI (Alfatest, Rome, Italy). Before each imaging session, bead slide calibration was performed for aligning the channels, to achieve a channel mapping precision smaller than 12 nm. Images were taken in dSTORM mode using 50% laser power for the 647 nm channel, 30% laser power for the 488 nm laser channel, and 30% for the 555 channel. Three-channels (2000 frames per channel) (647, 555 and 488) were acquired sequentially at 30 Hz (Hertz) in total reflection fluorescence (TIRF) mode. Single-molecule data was filtered using NimOSsoftware (v.1.18.3, ONI) based on the point spread function shape, photon count and localization precision to minimize background noise and remove low-precision and non-specific colocalization. Data has been processed with the Collaborative Discovery (CODI) online analysis platform https://www.alto.codi.bio/ from ONI and the drift correction pipeline version 0.2.3 was used. Clustering analysis was performed on localizations and BD clustering-constrained parameters were defined (photon count 300-max, sigma 0–200 nm, p-value 0–1, localization precision 0–20 nm). Colocalization was defined by a minimum number of localizations for each fluorophore/protein within a distance of 100 nm or a distance used from the centroid position of a cluster.

### Cytofluorimetric analysis

MACSPlex Exosome Kit (Miltenyi Biotec, Bergisch Gladbach, Germany) containing fluorescent labeled (FITC-PE) capture beads coupled to 37 exosomal surface epitopes and 2 isotope controls was used, following the manufacturer’s instructions (in detail: CD3, CD4, CD19, CD8, HLA-DR, CD56, CD105, CD2, CD1c, CD25, CD49e, ROR1, CD209, CD9, SSEA-4, HLA-ABC, CD63, CD40, CD62P, CD11c, CD81, MCSP, CD146, CD41b, CD42a, CD24, CD86, CD44, CD326, CD133-1, CD29, CD69, CD142, CD45, CD31, REA control, CD20, CD14, mIgG1 control). Briefly, 15 μL of beads were added to 120 μL of buffer or sample, including a total of 1 × 10^9^ EVs, and the complex was then incubated on a rotor overnight at 4 ℃. After the incubation and washing steps, a cocktail of APC fluorescent antibodies against tetraspanins (CD9, CD63 and CD81) was added (allowing the detection of beads bound EVs) and set on the rotor for 1 h at room temperature. After washing, samples were detected using BD FACSCelestaTM Flow Cytometer (BD Bioscience, NJ, USA). Median background values of buffer control were subtracted, and samples were normalized to the median fluorescence intensity of tetraspanins.

### Western blot analysis of cytoplasmic markers

Proteins extracted from Hela cells were used as cellular control, the pellet was resuspended in RIPA buffer (50 mM Tris–HCl, pH7.4, 150 mM NaCl, 1% Triton X-100, 1% Na-deoxycholate, 0.1% SDS, 0.1 mM CaCl_2_, and 0.01 mM MgCl_2_ supplemented with protease inhibitor cocktail (Thermo Fisher Scientific), incubate 30 min in ice vortexing every 10 min and centrifuge 20 min at 20,000 × *g*. An equal volume of bioproducts (38 µL) was loaded and separated on 4–15% Mini-PROTEAN TGX Precast Gels (Bio-Rad, USA). Bioproducts and cell lysates were treated with protein loading dye (Laemmli sample buffer; Bio-Rad) with freshly added β-mercaptoethanol 10%; v/v; Sigma, Germany) and boiled for 5 min at 95 ℃ before SDS-PAGE. Proteins were subsequently blotted to a nitrocellulose blotting membrane (0.2 µm; 1,060,000; GE Healthcare, USA). Membranes were blocked in 5% BSA (Carl Roth, Germany) in 0.1% Tween in TBS (TBS-T). After blocking, blots were probed with the following primary antibodies diluted in 5% BSA/TBS-T: Calnexin (1:500 dilution, E-10, Santa Cruz Biotechnology). After overnight incubation at 4 ℃, membranes were washed 3 times with TBS-T and subsequently incubated with the secondary antibody dilution: Polyclonal Goat anti-mouse HRP (1:5000 dilution; P0447) for 1 h at room temperature followed by washing. Blots were then developed using Western Bright ECL (541,004; Biozym Scientific, Germany) and protein bands were detected using the FusionCapt Advanced Solo 4 (Vilber, Germany).

### PBMC proliferation assay

The capacity of ASC or their bioproducts to inhibit induced proliferation of peripheral blood mononuclear cells (PBMCs) was analyzed as described before [27]. PBMCs were isolated from leukapheresis samples from healthy donors, provided by the German Red Cross Blood Donor Service in Mannheim (Mannheim Ethics Commission; vote number 2018-594 N-MA). To assess their proliferation, PBMCs were labelled with proliferation dye Cytotell Green (ATT Bioquest, 22,253) (1:500 dilution) and seeded at a 1:10 ASC/bioproduction:PBMCs ratio in RPMI, supplemented with 10% FBS, 2% l-glutamine (PAN Biotech, P04-80100), 1% Penicillin/Streptomycin (PAN Biotech, P06-07100), and 200 U/mL IL-2 (Promokine, C61240). PBMC proliferation was stimulated with phytohemagglutinin-L (PHA, 4.8 µg/mL (Biochrom, Merck Millipore, M5030)). PBMCs cultured alone without ASC in the absence and presence of PHA served as negative and positive controls, respectively. After 5 days, PBMC proliferation was measured based on the dilution of Cytotell Green dye using a FACS Canto II (BD Biosciences) and the data were analyzed with FlowJo Software.

### Phagocytosis

THP-1 monocyte-like cell line (ATCC, Manassas, VA, USA) were cultured in RPMI-1640 growth medium with l-Glutamine (Sigma-Aldrich Ireland Ltd. Wicklow, Ireland) supplemented with 10% FBS (Sigma-Aldrich), 1% penicillin G (100 U/mL) and streptomycin (100 μg/mL) solution (Sigma-Aldrich). In vitro assessment of phagocytic activity was done as described before [30]. THP-1 cells were seeded at a density of 5 × 10^4^ cells/well in dark 96 well-plates (Perkin Elmer Ireland Ltd. Dublin, Ireland) and exposed to 1 µg/mL of para-methoxyamphetamine (PMA, Sigma-Aldrich, St. Louis, MO, USA) for 48 h to induce a macrophage-like phenotype. Cultures were washed with DPBS and fed with growth media for 24 h. Afterwards, cells were activated with 100 ng/mL of lipopolysaccharide (LPS, Sigma-Aldrich) for 24 h. To measure the phagocytic capacity, Zymosan A FITC BioParticles^™^ (Thermo Fisher Ltd.) were used. Particles were opsonized with human serum (2 mg/mL per 2 × 10^7^ particles, Sigma-Aldrich) for 1 h and added to the cells in experimental media containing ASC bioproducts and growth media for 4 h. Then, cells were washed twice with DPBS, fixed with 4% PFA for 15 min and stained with Hoechst 33,342 (Invitrogen, Thermo Fisher Ltd). Images were taken on the Cytation 1 Imaging Reader at 20X (BioTek, with Gen5 Version 3.04 software, Swindon, UK). Six replicates were undertaken per condition and particle analysis was done by counting particle opsonization in a minimum of 200 cells per well.

### Cell migration assay on HUVEC

HUVEC were seeded in 48-well plates at 84,000 cells/cm^2^ and cultured overnight. Subsequently, a p200 tip was used to create a scratch in each monolayer. Cultures were washed with DPBS before adding EVs/CM as described before. Complete EndoGRO-LS medium was used as a positive control, while EndoGRO-LS without FBS and VEGF served as a negative control. Scratches were imaged immediately after the addition of CM (0 h) and after 8- and 24-h incubation using the automated Cytation 1 Imaging Reader at 4X. Six replicates were undertaken, and the total area of each scratch was measured using Image J. The percentage of closure was calculated relative to time 0 h.

### Cell migration assay on ASC

20,000 ASC were seeded in a 96-well Essen ImageLock^™^ plate and cultured overnight. Then, a 96-pin WoundMaker was used to create precise and reproducible wounds in all the wells. After the wound, the cells were washed 2 times with DPBS and ASC bioproducts added in different concentrations. Plates were then cultured in an IncuCyte ZOOM^™^ incubator and every 3 h were taken a picture with the software. The results were analyzed after 24 h. Relative Wound Density algorithm was used to report data.

### Angiogenesis

Human umbilical vascular endothelial cells (HUVEC) either from Lonza or prepared as described before [31] and cultured until the 6th passage in EndoGRO-LS Complete Culture Media Kit (SCME001, Sigma-Aldrich, St. Louis, MO, USA). In vitro formation of capillary-like structures was performed on growth factor–reduced Matrigel (356,231, Corning, NY, USA, center 1 and 3) or geltrex (Geltrex^™^ LDEV-free reduced growth factor matrix; Thermo Fisher Scientific, United States, center 2) HUVEC cells were treated with EVs or CM as described before, seeded at a density of 10 × 10^3^cells/well on a 48-well plate. Positive control was full EndoGro-LS medium, negative control medium without VEGF and FBS (as used for all the conditions). Cells were periodically observed with a Nikon TE2000E inverted microscope (Nikon, Tokyo, Japan), and experimental results were recorded after 16 h; 3 images were taken per well. Image analysis was performed with the ImageJ software v.1.53c, using the Angiogenesis Analyzer (center 1,3). The data from three independent experiments were expressed as the mean ± SD of tube length in arbitrary units per field. Center 2 used live cell imaging (Incucyte Zoom) to assess network formation as described before [32].

### VEGF quantification

Presence of vascular endothelial growth factor (VEGF) on ASC bioproducts was determined by solid phase sandwich ELISA using the human VEGF DuoSet ELISA (R&D Systems, USA) according to manufacturer’s instructions. The samples were read immediately at 450 nm with a wavelength correction at 570 nm using a VICTOR X4 multilabel plate reader (Perkin Elmer, Waltham, Massachusetts, USA). Levels of cytokines were quantified against an eight-point standard curve using twofold serial dilutions in reagent diluent.

### Protein content analysis

The Pierce^™^ BCA Protein Assay Kit (ThermoFisher Scientific, UK) was used to determine protein concentration. In order to quantify the total amount of protein, samples were first lysed with RIPA buffer 4:1 (ThermoFisher Scientific, UK) for 30 min on ice. The assay was carried out as per manufacturer’s instructions. The absorbance values were read in a VICTOR X4 plate reader (Perkin Elmer) at a 550 nm wavelength, and the protein concentrations of the samples were quantified against the standard curve.

### Statistical tests

Statistical analysis was performed using GraphPad prism v9.4.2 (GraphPad software, USA). Data are expressed as mean ± standard deviation (SD). ‘N’ indicates biological replicates; ‘n’ indicates technical replicates. Statistical differences among groups were calculated using ordinary two-way analysis of variance (ANOVA) and Tukey’s post-hoc test when group distributions were normal (Shapiro–Wilk’s test) and variances of populations were equal (Bartlett’s test). When either or both assumptions were violated, non-parametric analysis was conducted; Kruskal–Wallis test used to perform multiple comparison analysis and Dunn’s multiple comparison test for pairwise comparison. Results were considered statistically significant when p > 0.05.

## Results

### EV isolation

To discern the effects of production and isolation on EV phenotype and functionality, two main methodologies were followed: ultracentrifugation (UC) and size-exclusion chromatography (SEC). Throughout the manuscript the following terminology is used: EV isolated by ultracentrifugation were named EV-UC (Fig. 1A) while EV isolated by SEC were named EV-SEC (Fig. 1B). Additionally, the protein-rich supernatant obtained after SEC was named protein-rich fraction (Fig. 1B). The whole secretome or conditioned media (CM), collected as a comparator, was named CM thereafter (Fig. 1C) and the wash-off CM-WO, respectively.

### Impact of isolation method on EV phenotype

Figure 1 describes how each ASC bioderived product was produced and further distributed. Thus, in each center, experiments were performed comparing the complete array of ASC bioproducts.

We first differentially characterized EVs obtained by UC or SEC from three different ASC donors. Both EV preparations as well as the conditioned media (CM) counterpart were first measured by nanoparticle tracking analysis (NTA) in two of the expert centers using either Nanosight or Zetaview NTA, with established in house protocols. Particle concentration per cell was comparable between donors and within one method (Table 1A, Additional file 1: Fig.S1). EV-SEC, protein-rich fraction and CM samples showed similar particle concentration among them and between centers. Despite inter-method variability, the particles detected were all within the expected size range of extracellular vesicles (70 nm–300 nm), with EV preparations showing a population with a smaller mean particle diameter of 128.98 and 167.2 nm compared to the protein-rich fraction with 134.17 and 246.93 nm respectively (Table 1B).

**Table 1:** The nanoparticle tracking analysis of EV-UC, EV-SEC, Protein-rich fraction, CM and CM-WO samples each derived from 3 different ASC donors

A. No particles/10^6^ cells					
ASC	EV-UC	EV-SEC	Protein rich fraction	CM	CM-WO
NanoSight					
Donor 1	6.38E + 06	5.79E + 08	1.06E + 08	1.57E + 07	1.06E + 08
Donor 2	9.75E + 06	3.28E + 07	2.54E + 07	1.28E + 07	3.40E + 07
Donor 3	4.67E + 06	2.35E + 08	1.65E + 08	1.81E + 07	2.88E + 07
ZetaView					
Donor 1	2.13E + 08	7.72E + 08	7.72E + 07	2.10E + 08	0
Donor 2	7.08E + 08	7.83E + 08	2.17E + 07	3.00E + 08	0
Donor 3	3.17E + 08	1.52E + 09	4.78E + 08	3.00E + 08	0

B. Particle diameter (nm)					
ASC	EV-UC	EV-SEC	Protein rich fraction	CM	CM-WO
NanoSight					
Donor 1	123.6	–	135.9	98.0	165.5
Donor 2	128.8	99.2	141.8	98.1	–
Donor 3	113.7	179.6	124.8	160.3	122.9
ZetaView					
Donor 1	139.7	119.4	209.0	136.4	0
Donor 2	190.2	308.5	273.2	118.8	0
Donor 3	126.2	119.6	258.6	114.4	0

The analysis was performed on samples derived from cell passage 4. **A** Number of particles derived from 10^6^ ASC. **B** Mean particle diameter in nm. Data are shown as the mean of N = 3 ASC donors

ASC - adipose stromal cells

EV-UC - Extracellular vesicles isolated by ultracentrifugation

EV-SEC - Extracellular vesicles isolated by size exclusion chromatography

CM - Conditioned medium

CM-WO - Conditioned medium wash-off

**Fig. 2:**
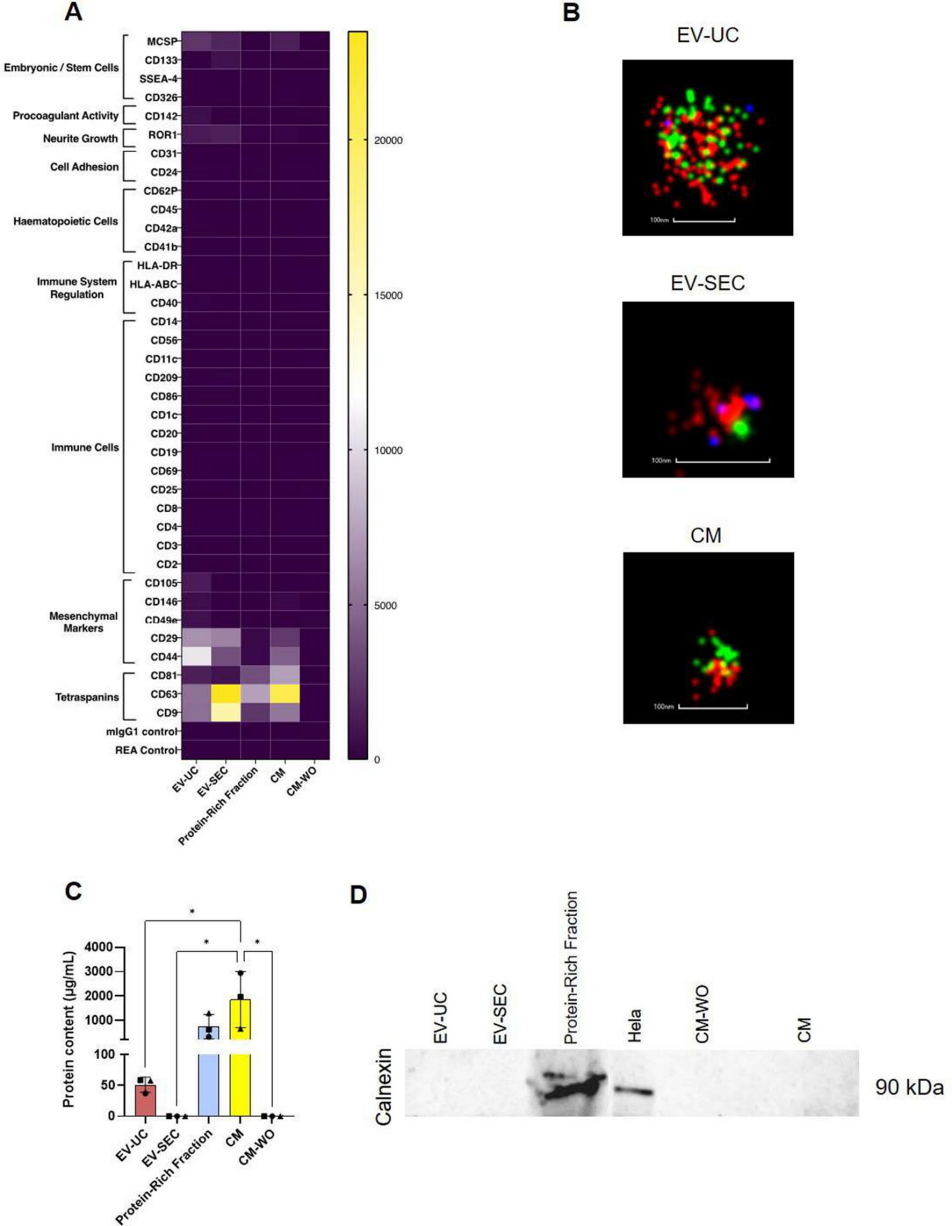
Characterization of the ASC bioproducts. **A** FACS analysis using the MACSPlex exosomal kit. EV-UC, EV-SEC, Protein-rich fraction, CM and CM-WO were tested for 39 surface markers divided into groups based on their origin and function (embryonic cells, pro-coagulation activity, neurite growth, cell adhesion, hematopoietic cells, immune system regulation, immune cells, mesenchymal cells, tetraspanins and two controls). Data represented as a heatmap with the mean fluorescence intensity of N = 3 ASC donors per marker. **B** Representative super-resolution microscopy pictures of EV-UC, EV-SEC, CM. The samples are stained with CD81 red, CD44 green and CD9 blue. The scale bar is 100 nm. **C** Protein content analysis showed higher concentration of protein in CM and Protein-Rich Fraction preparations and lower in EV-UC. No protein content was detected in the EV-SEC or the CM-WO groups. **D** Western blot analysis of Calnexin of EV-UC, EV-SEC, Protein-rich fraction, CM-WO, CM. Cell lysates of Hela cells were used as a positive control

To better understand the composition of the EV populations, samples were analyzed by flow cytometry (Fig. 2A). Importantly, this analysis showed no EV marker expression in the CM-WO, demonstrating the non-specificity of the particle count obtained by the nanodrop NTA analysis with these samples. Tetraspanins CD9 and CD63 were higher expressed in EV-SEC and the CM compared to UC-EV. The protein-rich fraction showed higher expression of tetraspanins than EV-UC. CD81 was weakly positive in CM, followed by the protein-rich fraction, while EV were negative. MSC marker CD44 and CD29 were detected in both EV preparations and at a lower level in CM but were negative in the protein-rich fraction. Other markers such as CD49e, CD146 and CD105 were almost exclusively present on the EV-UCs (Fig. 2A). Analyzing the immunological markers, we observed similar levels of CD29, MCSP, ROR1 for all bioproducts. CD142 (tissue factor, TF) was only present in EV-UC and CD133-1 on EV-SEC. Detailed relative fluorescence intensity measurement of the main markers are shown in Additional file 1: Figure S2.

Characterization of the surface markers and particle size was confirmed by super-resolution microscopy, staining for the tetraspanins CD9 (blue) and CD81 (red), and the MSC marker CD44 (green). Overall, all particles showed CD44 expression and differential tetraspanin expression, as EV-SEC preparations showed different marker composition compared to EV-UC and CM. Most particles were single tetraspanin positive, with EV-UC and CM being mainly CD81 positive and EV-SEC CD9 positive (Fig. 2B).

Protein concentration was higher in CM and protein-rich fraction and low in the EV-UC and non-detectable in EV-SEC and CM-WO (Fig. 2C). Absence of subcellular components that might have been contaminating the EV preparations was confirmed by western blot analysis of the cytoplasmic marker Calnexin, only detected in the protein-rich fraction and the positive control Hela cell lysate (Fig. 2D) (Additional file 3: Whole western blot membrane).

### Differential immunomodulatory activities of bioderived ASC products

The impact of the different bioproducts released by ASC, and of their preparation method, was first addressed by investigating the immunomodulatory properties as a key therapeutic effect of MSC. For this, and to overcome the calculation differences from the two NTA devices, a cell equivalent dose of each bioproduct was calculated to allow comparison.

**Fig. 3:**
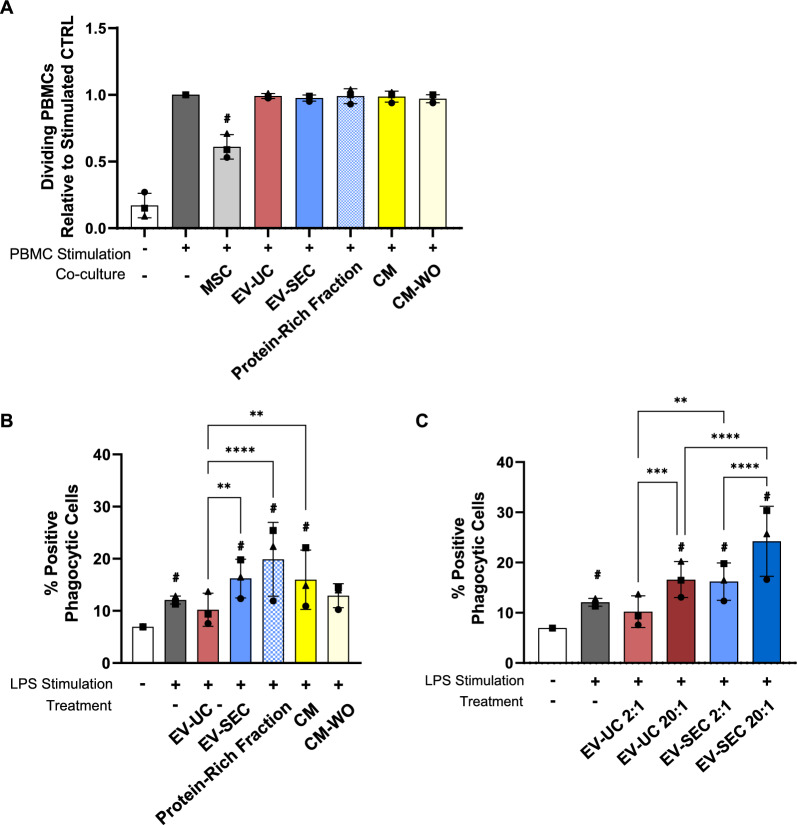
Immunomodulatory properties of ASC bioproducts. **A** PBMC proliferation after five days of co-culture with ASC under PHA stimulation. All values were normalized to PHA-stimulated monoculture PBMCs. **B** LPS-stimulated THP-1 cells showed an increase in their phagocytic activity when compared to non-stimulated cells (Neg Ctrl). Treatment with EV-SEC, Protein-Rich Fraction and CM enhanced the number of positive phagocytic cells with respect to the Neg Ctrl and the EV-UC group. **C** Increasing the ratio of EV:THP-1 cells to 20:1 significantly enhanced the phagocytic stimulation of both EV preparations. Data are represented as the mean ± SD of N = 3 ASC donors and n = 6 technical replicates. Statistical analysis was performed using Two-Way ANOVA with Tukey’s multiple comparison test; * = p < 0.05, ** = p < 0.001, *** = p < 0.0001, **** = p < 0.00001. # Significance versus the negative control

Confirming previous observations [27, 28], the proliferation of mitogen-stimulated PBMCs was exclusively inhibited by ASC being present in a direct coculture while no decrease in proliferation was seen when adding any of the EV preparations or the CM (Fig. 3A). This strongly suggested that the already previously observed lack of inhibitory activity [27, 28] was independent of the manufacturing mode of EVs, as both ultracentrifugation and SEC-isolated EVs lacked inhibitory potential. The flow-through resulted from the concentration step (thereafter named wash-off, CM-WO) was used as a control.

In contrast, differential effects were seen when assessing the phagocytic activity of THP-1 monocyte derived macrophage cells after LPS stimulation. The EV-SEC preparation together with the protein-rich fraction and the CM significantly enhanced the phagocytic activity compared with the negative control (non-stimulated cells) and were superior to the EV-UC preparation (Fig. 3B).

To assess whether the effect could be further increased, we raised the concentration tenfold to add 2 and 20 EVs:THP1 cell. The higher EV concentration significantly enhanced the efficacy of both EV preparations, 10%/16.6% for 1:2/1:20 for EV-UC and 16.2%/24.2% for EV-SEC (Fig. 3C).

### Effects of ASC bioproducts on cell migration

Next, we sought to investigate whether other key mechanistic properties of ASC such as the effect on cell migration were also influenced by the type and preparation of the different biofactors modulated by the methodology of EV isolation.

**Fig. 4:**
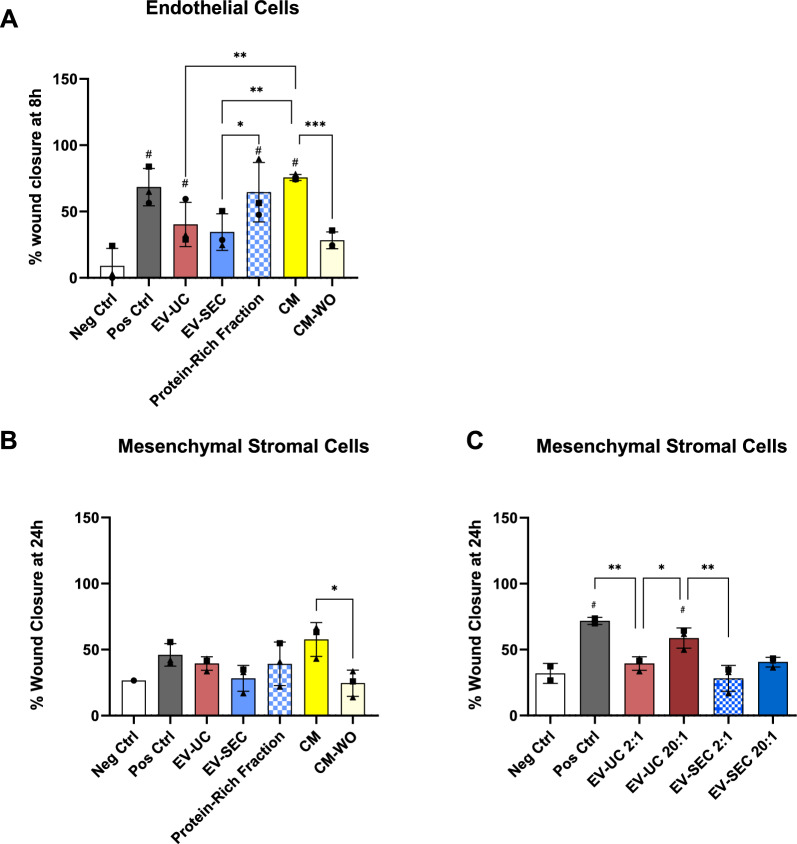
Enhancement of cell migration by ASC bioproducts. **A** Protein-Rich Fraction, CM and EV-UC preparations significantly enhanced endothelial cell migration with respect to the negative control (EndoGro-LS medium without FBS and VEGF, positive control with FBS and VEGF) in an in vitro wound healing model at 8 h after injury. CM and Protein-Rich Fraction displayed higher abilities to promote cell migration than any other treatment. **B** Migratory capacity of ASC 24 h after injury showed a tendency to increase after the addition of CM respect to the negative control (MEM-⍺ media without FBS) and positive control (MEM-⍺ media with FBS) (**C**) Increasing the ratio of EV:MSC leads to a significant increase in cell migratory capacity of EV-UC. Data are represented as the mean ± SD of N = 3 ASC donors and n = 6 technical replicates. Statistical analysis was performed using Two-Way ANOVA with Tukey’s multiple comparison test; * = p < 0.05, ** = p < 0.001, *** = p < 0.0001, **** = p < 0.00001. # Significance versus the negative control

The ability to influence cell migration was tested using the in vitro wound scratch assay on endothelial cells and ASC. CM and protein-rich fraction significantly enhanced endothelial cell migration after 8 h of injury induction to levels of the positive control (to eightfold and sevenfold compared to control, respectively) (Fig. 4A). To a lesser extent, the EV-UC population increased endothelial cell migration in the scratch assay (fourfold compared to control) while EV-SEC, but also CM-WO improved migration by fourfold and 3.1-fold.

A similar trend was seen when the bioderived products were applied to assess their impact on ASC migration. Although differences did not reach statistical significance, CM, protein-rich fraction, and EV-UC groups showed a trend to enhance ASC migration (Fig. 4B). EV-SEC, again, was not effective. Of note, CM-WO had significantly less potency to induce scratch wound healing compared to CM. Again, the stimulating capacity of EV-UC was dose-dependent, while EV-SEC at higher concentrations still exerted only a marginal increase in ASC migration (Fig. 4C).

### Effects of ASC bioproducts on angiogenesis

Having observed differences of ASC bioproducts and especially EVs prepared by different methods, we next tested their capacity to induce angiogenesis. To address whether the international shipment of samples may have hampered their potential therapeutic effects, this assay was run in the three centers participating in the study using their established in-house methods.

The angiogenic capacity of ASC bioproducts was measured by the ability to induce the formation of tubule-like structures in endothelial cells seeded on an extracellular matrix, either Matrigel^™^ or Geltrex^®^. Results were normalized to the negative control (serum-free medium) and compared among centers, VEGF added served as positive control.

**Fig. 5:**
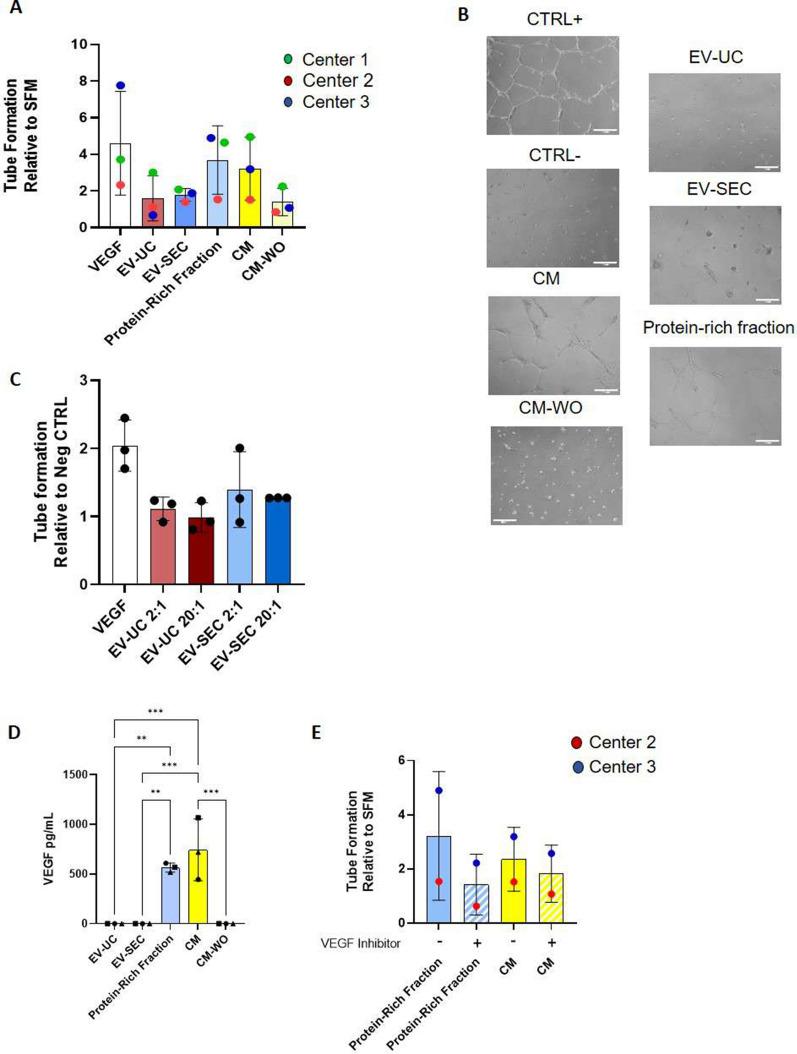
Angiogenic properties of ASC-derived bioproducts. Tube formation assay was chosen as inter-center comparison between centers. **A** Ability of ASC bioproducts to stimulate tube formation in vitro is represented as the relative tube formation of the negative control (EndoGRO-LS medium without VEGF). A wide range of tube formation was achieved in each center, with similar trends observed among groups, whereby CM and Protein-Rich Fraction showed superior angiogenic support abilities. **B** Representative phase contrast images of tubule-like networks in culture. **C** Increasing the EV ratio to 20:1 did not impact tube formation. **D** VEGF concentration was highly found in CM and protein-rich fraction but absent in both EV preparations. **E** Addition of 1 µM of ZM32381 in the CM and the protein-rich fraction preparations resulted in reduced tube formation. Data are represented as the mean ± SD of N = 3 ASC donors and n = 3 technical replicates per center. Statistical analysis was performed using One-Way ANOVA (D, E). * p < 0.05, ** p < 0.01, *** p < 0.001

Overall, the rates of angiogenesis induction showed comparable trends between centers. CM and protein-rich-fraction induced tube-like formation to the highest degree, comparable to levels induced by VEGF, whereas EV-UC and EV-SEC induced a moderate angiogenic effect (Fig. 5A, B). No dose-dependency on angiogenic induction capacities was seen when the EV doses were increased tenfold (Fig. 5C).

Based on these results, we sought to investigate the levels of VEGF present in each of the bioproducts which were assessed. VEGF was detected only in CM and protein-rich fraction samples, but not in the EV populations and the CM-WO (Fig. 5D), similar to the amount of total protein (Fig. 2C).

The addition of the selective VEGFR-2 inhibitor ZM323881 significantly reduced tube formation capacity of CM and protein-rich fraction (Fig. 5E), thereby confirming the critical role of VEGF in the induction of tubes.

## Discussion

In recent years, a growing body of literature has attributed the main therapeutic effects of MSC to their secretome, which represent a new and safer method of treating medical conditions than direct transplantation of cell-based therapies [12]. However, the term secretome includes a broad range of components with different nature that must be properly characterized and defined before reaching clinical translation.

In this study, we tested the in vitro efficiency of the different components of the ASC secretome on several aspects involved in tissue regeneration. To our knowledge, it represents one of the few studies characterizing and comparing EV obtained using different isolation methodologies and the protein-rich fraction to the whole secretome in order to identify the component that had the greatest efficacy in a variety of assays and in an interlaboratory approach.

Following the international guidelines [5], the bioproducts were first characterized by demonstrating the concentration and diameter, specific EV and intracellular markers, and the absence of cytoplasmic contaminations. It has previously been shown that the use of different NTA technologies can result in largely differing accuracy and precision [33], underlining the need for transparent reporting of experimental details and calculating cell equivalents rather than particle concentrations, especially for dose-effective studies. The different concentration of particles between the two EV preparations might be due to the different culture systems (2D flask vs bioreactor), as bioreactor production has recently been shown to enhance EV production [34]. Despite different levels of specific marker expression, both EV populations were consistent with the identification criteria. Further, as expected, the protein concentration was higher in the CM and the protein-rich fraction. While the lower concentration detected in the EV-UC was reasonable and comparable to previous published results [35], the low levels found in the EV-SEC preparation were surprising. We hypothesized that these results could reflect a technique limitation—the lower detection range of the technology used is 20 µg/mL—or a higher EV purity.

Two of the key roles of MSC in injury resolution and tissue healing are the ability to modulate the host’s immune system and promote angiogenic processes and tissue regeneration [36]. The role of EVs in regulating the immune system is still unclear and inconsistent. While there are studies showing that EV successfully suppress mitogen-induced T cell proliferation, others (ourselves included) failed to reproduce such observations [27, 28]. In the current study, ASC-derived bioproducts failed to inhibit mitogen-driven T cell proliferation regardless of the isolation methodology—much like previous data where neither EV nor MSC-secretome could suppress T cell proliferation despite the use of licensing strategies [27, 37]. Only in the presence of MSC, we observed a reduction in T cell proliferation, indicating that this mechanism requires cell-to-cell contact and indoleamine 2,3-dioxygenase (IDO) as molecular mediator of the T cell inhibition process [38]. Adding to these observations, Papait and colleagues recently reported that ASC-derived EV isolated by ultracentrifugation lacked the propensity to inhibit anti-CD3-driven T cell proliferation and to modulate Th1/Th2/Th17/Treg differentiation [33].

The mechanism whereby the MSC regulate macrophage activation appears to be different. Several studies have reported that both EV preparations [39] and MSC secretome [30] increased the phagocytic activity of macrophages. In our hands, the protein fraction showed the greatest effect in enhancing phagocytic activity. EV SEC-isolated preparations also stimulated particle engulfment and this activity was shown to be dose-dependent. The above mentioned study by Papait et al. also compared ASC-conditioned medium *in toto*, the EV-free and the EV-UC preparation on macrophage differentiation, demonstrating the ineffectiveness of the EV preparation in skewing macrophage polarization to either M1 or M2 or myeloid dendritic cell maturation despite the use of four different doses (100–10 µL with ab average EV concentration of 9.9 × 10^5^ EV/µL) [37].

When looking at the ability to stimulate cell migration, both EV preparations were less effective compared to the protein-rich fraction and the CM. Due to the large volume of papers reporting the ability of EV to induce cell migration in other cell types [40–42], we tested the bioproducts on the derivative tissue. These experiments indicated that administering EV-UC populations at a higher dose enhanced cell migration. The minimal improvement in wound closure seen in EV-SEC bioproducts, a theoretically purer population, even at a higher dose brings up the attribution of therapeutical effects to EV that may have been elicited by co-isolated factors rather than the EV themselves [26].

We also evaluated the angiogenic properties of the ASC bioproducts in an in vitro tubulogenesis assay [43]. As all centers had established this assay before, it was performed in the three centers participating in this study. Additionally, this allowed us to validate the efficiency of our samples after being distributed across the participant laboratories. Similar to our previous results, EV populations showed minimal tube formation, lower than the levels seen by the protein-rich fraction and the CM. It is important to highlight the prominent variability between sites. Some of the reasons behind the distinct levels of performance may rely on intrinsic experimental variability such as the use of different ECM-like substrates (matrigel and geltrex) and endothelial cells [44] from different donors. However, we cannot rule out the possibility that in addition shipment might have influenced the quality of the samples.

VEGF has been described to play a major role in the MSC-derived angiogenic properties [32, 45]. The higher levels of VEGF detected in the protein-rich fraction and the CM but absence in the EV preparations provide further evidence to explain the previous results. Confirming the crucial role of this growth factor in the formation of tubule-like structures, inhibition of VEGF in the protein-rich fraction and the CM decreased the angiogenic efficiency [32]. Further, an increasing number of reports raise the awareness that at least some data demonstrating EV bioactivity may have been flawed by substantial contamination with soluble non-EV factors [25, 26, 46]. Whittaker et al. for instance showed that EV were only effective in stimulating angiogenesis and wound healing when used at low purity or very high concentrations [26]. Vice versa, contaminating factors could suggest negative effects of EVs, like those reported by Forteza-Genestra et al. in a model of chondrocyte differentiation where impurities caused deleterious effects on extracellular matrix component expression in chondrogenic cells [25].

Although our initial experimental design aimed to study the role of EV isolation methodology on the therapeutic potential in a specific application, the current results open a broader discussion. It is important to stress that the efficacy of an EV-based therapy relies on the successful uptake of the vesicles, a process highly dependent on the molecules present at a surface level as well as in the acceptor cells [47]. Based on our current knowledge, the ultimate functionality of the EV preparations is likely to be driven not exclusively in terms of purity—thus, the isolation method—but by the dose utilized. Supported by the results presented in this study, at least in certain applications, increasing the EV dosage can increase effectivity. In order to achieve clinically relevant doses, bioproduction needs to be highly scalable and time efficient.

## Conclusions

The data presented in this study adds to the current literature supported by others [48] and positions the significant advantages of using the whole secretome compared with the EV populations in key aspects of the MSC regenerative spectrum thanks to its increased immunomodulation abilities and superior angiogenic properties.

### Supplementary Information

**Additional file 1:** **Figure S1. **Characterization of the ASC bioproducts. Nanoparticle tracking analysis graphs show the size distribution of the isolated EVs obtained in two centers.

**Additional file 2:** **Figure S2.** Surface characterization of the ASC bioproducts. Surface markers expression from FACs analysis divided into tetraspanins (**A**), Immune markers (**B**), Mesenchymal markers (**C**). The samples were normalized to the median fluorescence intensity of tetraspanins.

**Additional file 3: Figure S3. **Whole western blot membrane.

## Data Availability

All data generated or analysed during this study are included in this published article [and its supplementary information files].

## Abbreviations

ASC: Adipose stromal/stem cells
CM: Conditioned medium
CM-O: Wash-off
EV: Extracellular vesicles
FBS: Fetal Bovine Serum
HGF: Hepatocyte growth factor
HUVEC: Human umbilical vascular endothelial cells
MSC: Mesenchymal stromal cells
NTA: Nanoparticle tracking analysis
PBMCs: Peripheral blood mononuclear cells
PBS: Dulbecco’s phosphate-buffered saline
SEC: Size-exclusion chromatography
TBS-T: Tween in TBS
TGF-β: Transforming growth factor β
UC: Ultracentrifugation
VEGF: Vascular endothelial growth factor
