# Supplementary material for: Adipose stromal cells bioproducts as cell-free therapies: manufacturing and therapeutic dose determine in vitro functionality
Source: J Transl Med. 2023 Oct 16;21:723. doi: 10.1186/s12967-023-04602-9 (PMC10577984; doi:10.1186/s12967-023-04602-9)
Supplement: Supplementary file 1 — Additional file 1: Figure S1. Characterization of the ASC bioproducts. Nanoparticle tracking analysis graphs show the size distribution of the isolated EVs obtained in two centers. [file 12967_2023_4602_MOESM1_ESM.pdf]

Centre 1

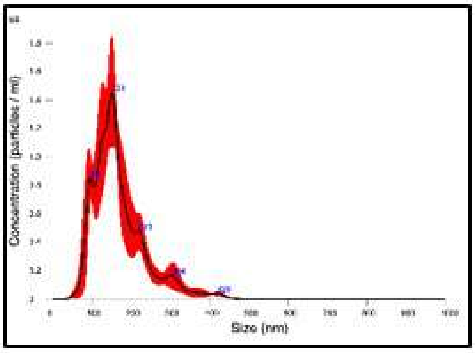

EV-UC

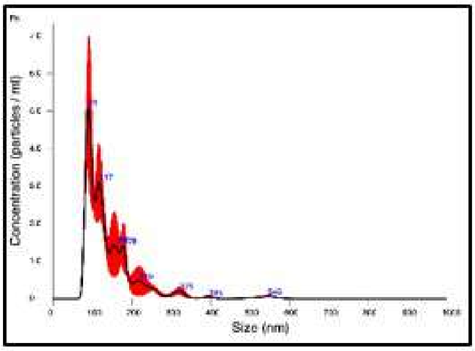

EV-SEC

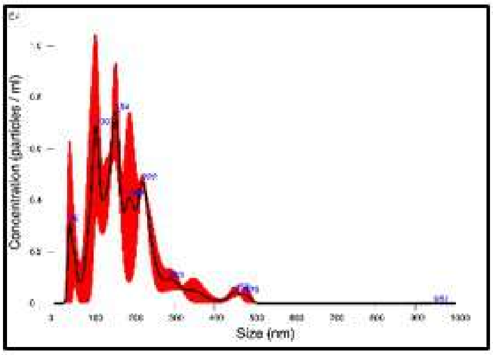

Protein-rich fraction

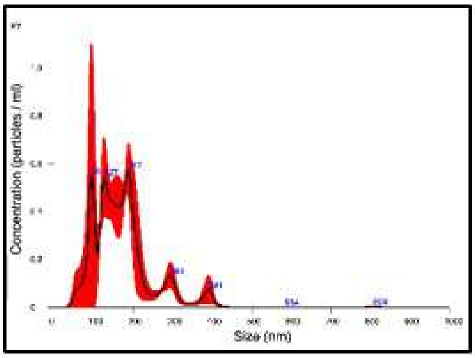

CM

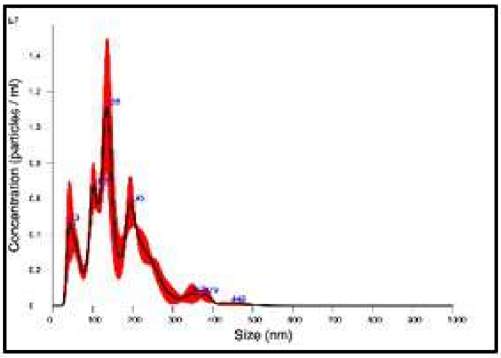

CM WO

Centre 2

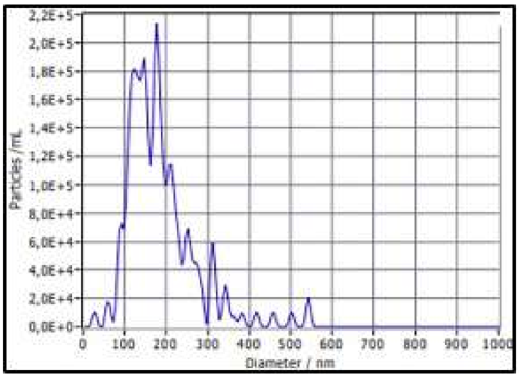

EV-UC

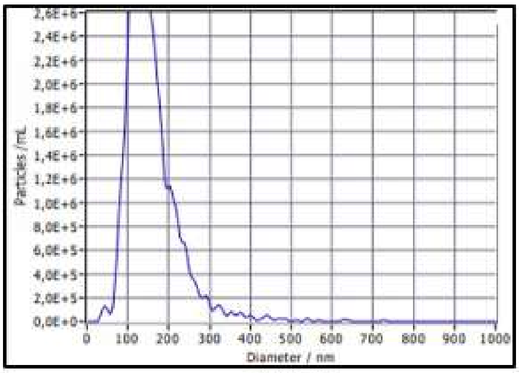

EV-SEC

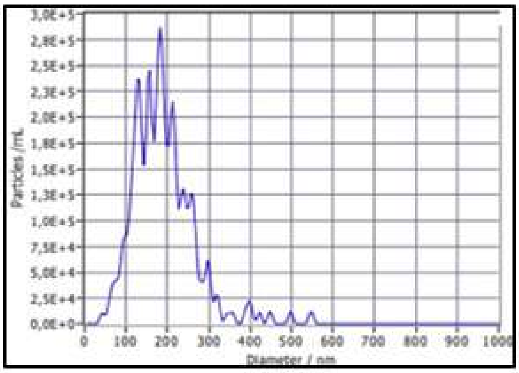

Protein-rich-fraction

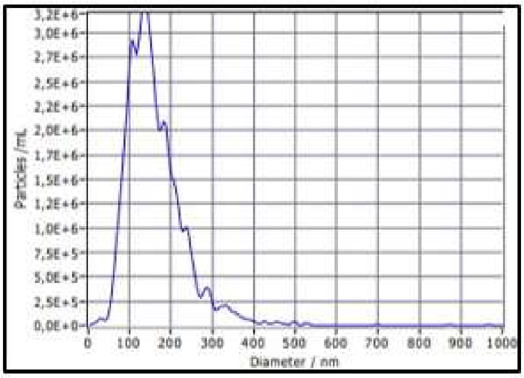

CM

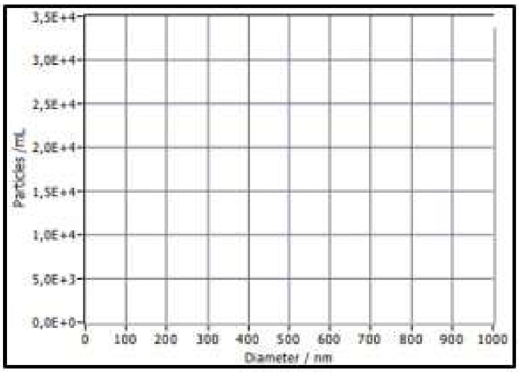

CM-WO
