# Supplementary figures and images for: Adipose stromal cells bioproducts as cell-free therapies: manufacturing and therapeutic dose determine in vitro functionality
Source: J Transl Med. 2023 Oct 16;21:723. doi: 10.1186/s12967-023-04602-9 (PMC10577984; doi:10.1186/s12967-023-04602-9)

### A Tetraspanins

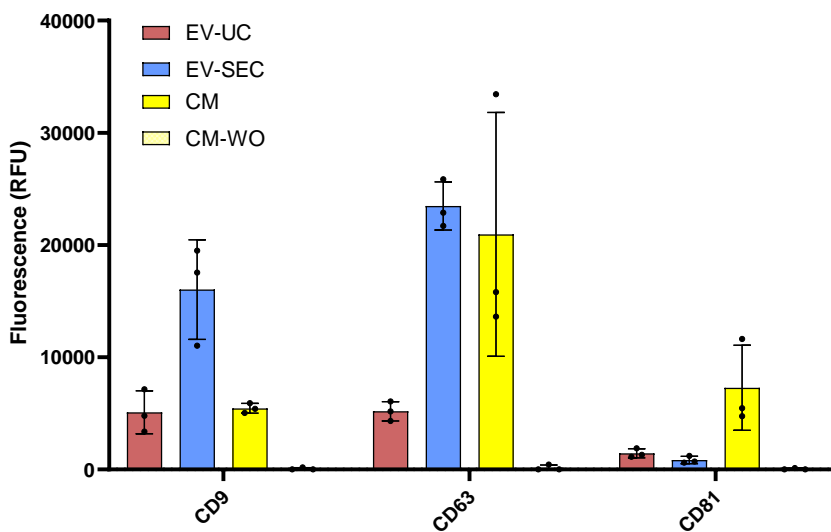

### B Immune Markers

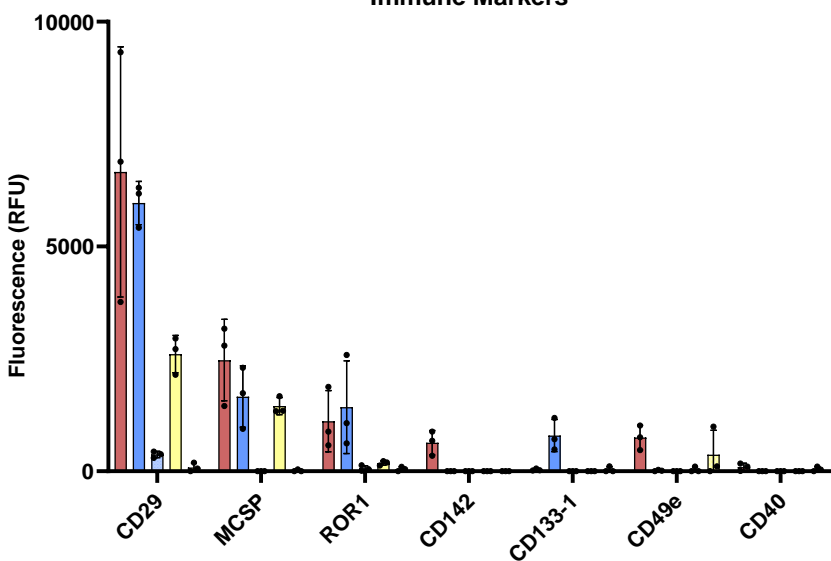

### C Mesenchymal Markers

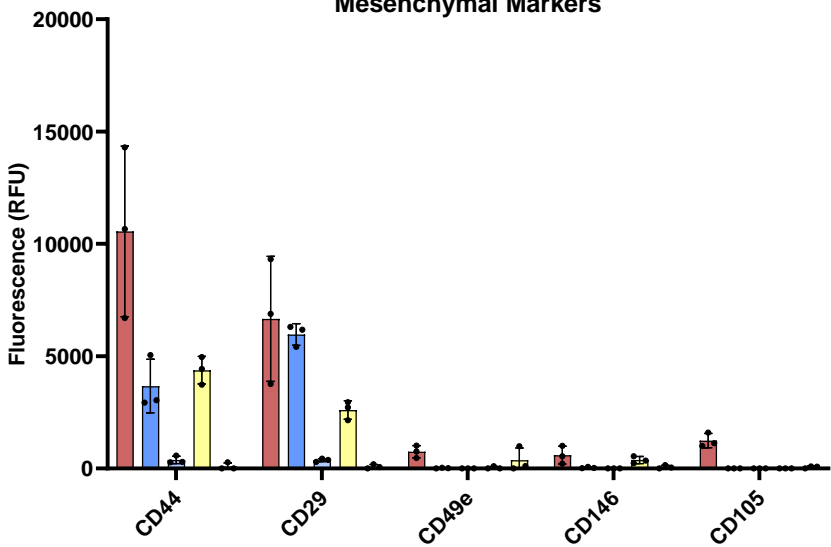

Supplement: Supplementary file 2 — Additional file 2: Figure S2. Surface characterization of the ASC bioproducts. Surface markers expression from FACs analysis divided into tetraspanins (A), Immune markers (B), Mesenchymal markers (C). The samples were normalized to the median fluorescence intensity of tetraspanins. [file 12967_2023_4602_MOESM2_ESM.pdf]

Marker

MSC

EV-UC

EV-SEC

Protein-Rich Fraction

Marker

Hela

CM-WO

CM

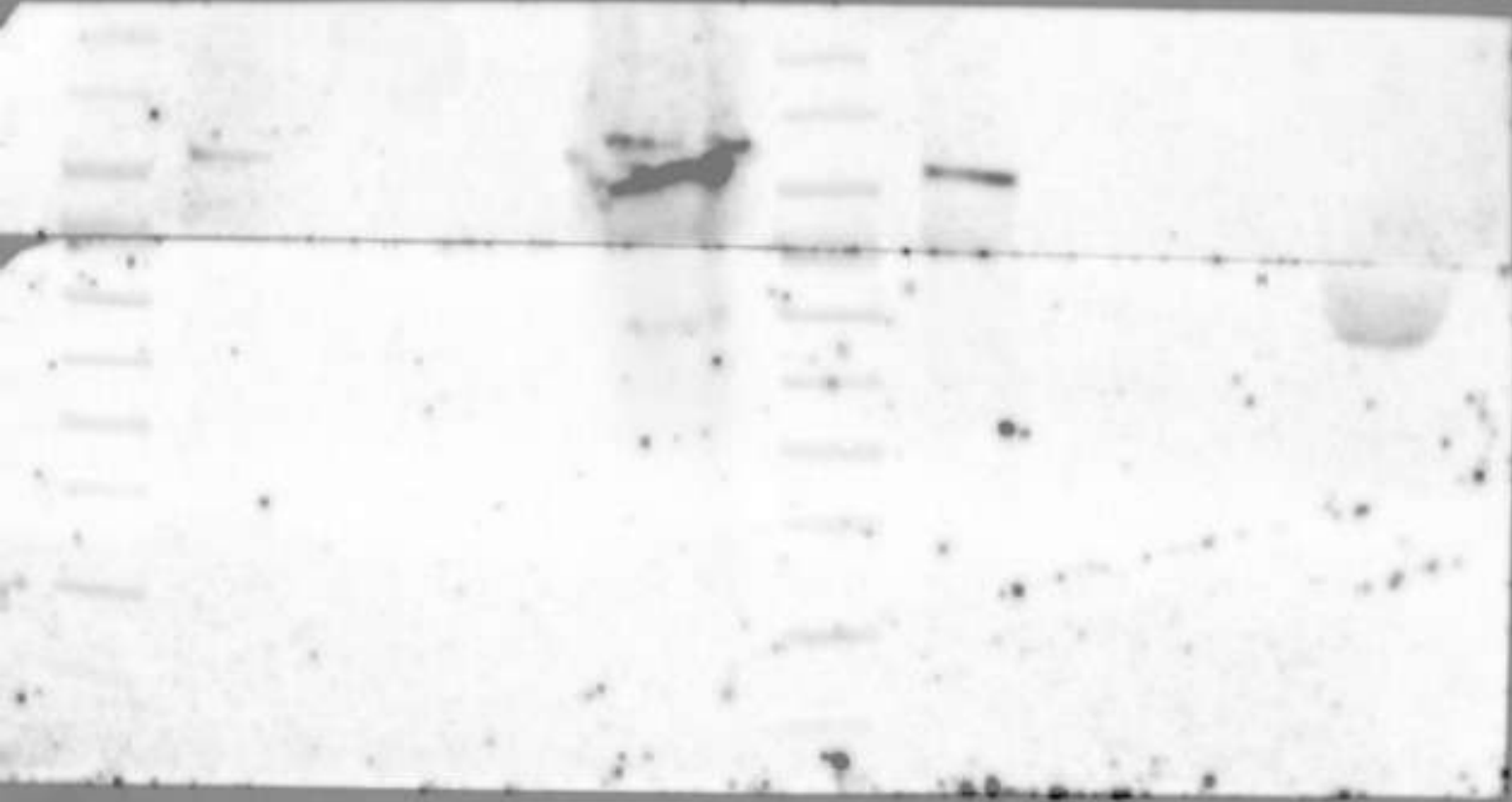

Supplement: Supplementary file 3 — Additional file 3: Figure S3. Whole western blot membrane. [file 12967_2023_4602_MOESM3_ESM.pdf]
